# Muscle Wasting in Hemodialysis Patients: New Therapeutic Strategies for Resolving an Old Problem

**DOI:** 10.1155/2013/643954

**Published:** 2013-12-05

**Authors:** Chun-Ting Chen, Shih-Hua Lin, Jin-Shuen Chen, Yu-Juei Hsu

**Affiliations:** Division of Nephrology, Department of Internal Medicine, Tri-Service General Hospital, National Defense Medical Center, No. 325, Section 2, Cheng-Gong Road, Neihu, Taipei 114, Taiwan

## Abstract

Muscle wasting has long been recognized as a major clinical problem in hemodialysis (HD) patients. In addition to its impact on quality of life, muscle wasting has been proven to be associated with increased mortality rates. Identification of the molecular mechanisms underlying muscle wasting in HD patients provides opportunities to resolve this clinical problem. Several signaling pathways and humeral factors have been reported to be involved in the pathogenic mechanisms of muscle wasting in HD patients, including ubiquitin-proteasome system, caspase-3, insulin/insulin-like growth factor-1 (IGF-1) signaling, endogenous glucocorticoids, metabolic acidosis, inflammation, and sex hormones. Targeting the aforementioned crucial signaling and molecules to suppress protein degradation and augment muscle strength has been extensively investigated in HD patients. In addition to exercise training, administration of megestrol acetate has been proven to be effective in improving anorexia and muscle wasting in HD patients. Correction of metabolic acidosis through sodium bicarbonate supplements can decrease muscle protein degradation and hormone therapy with nandrolone decanoate has been reported to increase muscle mass. Although thiazolidinedione has been shown to improve insulin sensitivity, its role in the treatment of muscle wasting remains unclear. This review paper focuses on the molecular pathways and potential new therapeutic approaches to muscle wasting in HD patients.

## 1. Introduction

Hemodialysis (HD) is a life-saving replacement therapy for patients with end-stage renal disease (ESRD). Although HD can maintain and even extend an acceptable quality of life for patients diagnosed with ESRD, long-term HD contributes to a number of serious complications, including cardiovascular disease, bleeding tendencies, renal osteodystrophy, gonadal dysfunction, protein malnutrition, insulin resistance, immunodeficiency, anemia, and muscle wasting [1]. Despite great advances in managing HD-related complications, muscle wasting is still an unresolved concern. Muscle wasting is defined as unintentional body weight loss, which can be divided into loss of lean body mass and fat mass, and has been recognized as a common and major problem of chronic kidney disease (CKD) that affects patient mortality rates, daily activity, quality of life, immunity function, and numbers of days of hospitalization [2]. In the general population, high body mass has been demonstrated as a conventional risk factor for cardiovascular events and all-cause mortality [3]. By contrast, previous studies have revealed a survival benefit in HD patients possessing high body mass, and patients on maintenance HD have been reported to suffer more severe muscle wasting than predialysis CKD patients [4]. Therefore, early recognition and treatment of muscle wasting are crucial for CKD patients to improve their quality of life and prognosis.

Muscle wasting in HD patients has been said to occur through either accelerated protein degradation or decreased protein synthesis. Protein homeostasis between synthesis and degradation depends on protein intake and utilization. Approximately 4 g of protein/kg of body weight is synthesized and degraded per day in a normal adult [5]. Skeletal muscle is a dynamic organ and is the largest reservoir of protein, which consists of amino acids and carbon chains. Muscle mass represents the most reliable indicator of protein homeostasis and is affected by a variety of clinical catabolic illnesses such as stress, liver failure, cancer, sepsis, diabetes, and CKD [6]. Accelerated protein degradation without a sufficient protein supply may lead to skeletal muscle atrophy resulting in muscle wasting. It has been shown that by measuring the turnover of labeled amino acids, the rate of protein degradation exceeds synthesis during and after HD in ESRD patients [7]. Recent advances in research on molecular mechanisms involved in the development of muscle wasting in HD patients have provided opportunities to resolve this old problem. In this review paper, we focus on recent advances in research on molecular mechanisms and the development of new therapeutic strategies for muscle wasting in HD patients.

## 2. Mechanisms of Muscle Wasting in Hemodialysis Patients

**Figure 1:**
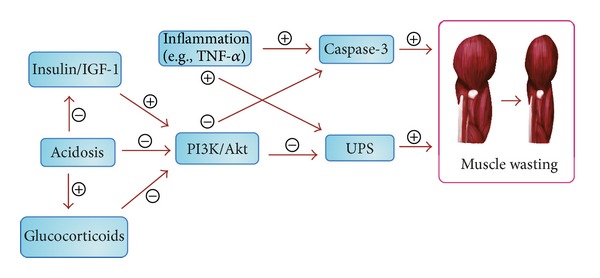
Molecular mechanisms and signaling pathways of muscle wasting in hemodialysis patients. UPS, ubiquitin-proteasome system; IGF-1, insulin-like growth factor-1; PI3K, phosphoinositide 3-kinase; TNF-*α*, tumor necrosis factor-*α*.

Muscle wasting is determined by complex mechanisms and several of these have been documented to explain muscle wasting in CKD patients with and without HD (Figure 1).

### 2.1. Ubiquitin-Proteasome System

Ubiquitin-proteasome system (UPS), the major mechanism of proteolysis, is activated in skeletal muscle in CKD patients, as well as in patients with other chronic diseases. Protein degradation is increased mainly through activation and upregulation of the UPS [8]. Ubiquitin is a small protein, which becomes covalent by attaching to the *ε*-amino group of lysine residues in the substrate proteins. The first step of ubiquitin conjugation is activation of ubiquitin by the E1 enzyme. Activated ubiquitin is subsequently transferred to the E2 enzyme, which is the ubiquitin carrier protein. When activated ubiquitin is conjugated with the E2 enzyme, it can be recognized by the E3 enzyme, which is ubiquitin protein ligase. The E3 enzyme catalyzes ubiquitin ligation to the substrate protein. The process is repeated to form a polyubiquitin chain, which is recognized and degraded by proteasome. In the system, proteasome is the major proteolytic enzyme that converts proteins to small peptides and amino acids. However, only polyubiquitinated proteins can be recognized and degraded by the proteasome. Proteins containing mono- or diubiquitin chains on sequential lysine residues are not recognized by the proteasome [9, 10]. When muscle protein degradation is initiated, the expression and activity of two muscle-specific E3 ligases, atrogin-1 (known as muscle atrophy F-box, MAFbx), and muscle-specific ring finger-1 (MuRF-1) are upregulated. The increase of activation of these ligases correlates with the acceleration of protein degradation. The forkhead transcription factors (FoxO) and the transcription factor nuclear factor kappa B (NF-*κ*B) have been identified as the regulatory factors of activation of E3 conjugating enzymes. The promoters for MAFbx and MuRF-1 are activated by FoxO and NF-*κ*B, respectively, leading to muscle protein degradation in the UPS [11, 12].

### 2.2. Caspase-3 Proteolytic Pathway

Caspase-3 is a protease that participates in cell apoptosis. It cleaves actomyosin in myofibrillar complexes and generates the 14 kDa actin fragment. Caspase-3 activation accelerates protein degradation in muscles. Elevated levels of 14 kDa actin fragment were revealed in muscle biopsies obtained from patients diagnosed with ESRD on maintenance HD or from those who had suffered a burn injury. The higher levels of 14 kDa actin fragment revealed active muscle wasting in patients with catabolic conditions and suggested that levels of the 14 kDa actin fragment can be used as a biomarker of muscle protein degradation [13, 14].

### 2.3. Insulin, Insulin-Like Growth Factor-1, and Insulin Resistance

Insulin is a major regulator in the modulation of protein synthesis and degradation in skeletal muscle. The metabolic effect of insulin on muscle protein turnover is characterized by suppression of protein degradation in a phosphatidylinositol 3-kinase (PI3K)/Akt-dependent pathway. Insulin binds to the insulin receptor (IR) on the cell membrane and activates the internal tyrosine kinase activity in cytosol. The insulin receptor substrate (IRS) proteins in cytosol are phosphorylated by activated IR. PI3K consists of p85 regulatory and p110 catalytic subunits and becomes an activated enzyme after binding to the phosphorylated IRS proteins. The activated PI3K catalyzes the production of phosphatidylinositol (3,4,5) triphosphate, which activates the serine kinase Akt by phosphorylation. Phosphorylated-Akt (p-Akt) affects a variety of regulators involved in metabolic processes in skeletal muscle. Decreased p-Akt activity stimulates the expression of E3 conjugating enzymes, atrogin-1/MAFbx, and MuRF1 in muscles. Activated IRS also activates the MEK/ERK mitogen-activated protein (MAP) kinase pathway, which is involved in the regulation of many critical biological processes, including cell proliferation, differentiation, and death [12, 15].

Insulin resistance leads to impaired insulin/IGF-1 signaling in skeletal muscle. Impaired insulin/IGF-1 signaling results in a decreased level of p-Akt in the muscle, which causes suppression of the PI3K/Akt pathway and muscle protein degradation. Accumulating evidence has demonstrated accelerated activation of the caspase-3 proteolytic pathway and a decreased level of p-Akt in the skeletal muscle in patients exhibiting insulin resistance, excess angiotensin II, inflammation, acidosis, and CKD [16, 17]. It has been well established that patients diagnosed with CKD suffer increased insulin resistance, which may contribute to muscle wasting [18].

Inflammation is also a major consequence of both CKD and HD, and numerous inflammatory mediators have been proven to modulate insulin-related signaling pathways in skeletal muscle. Inflammatory factors such as tumor necrosis factor-*α* (TNF-*α*) suppress insulin receptor signaling through the inhibition and degradation of IRS in skeletal muscle [19]. In addition, TNF-*α* activates caspase-3 and NF-*κ*B, which stimulates UPS activation, leading to muscle wasting [14, 20].

### 2.4. Glucocorticoids

The kidney normally excretes cortisol and its water soluble metabolites, and elevated serum cortisol levels have been reported in CKD patients because of the prolonged serum half-life of cortisol in advanced renal failure [21]. Glucocorticoids activate the glucocorticoid receptors, which can directly bind to the p85 subunit of PI3K, leading to muscle wasting by suppression of p-Akt. In addition, increased levels of the p85 subunit have been reported in CKD patients [22, 23]. Glucocorticoids also induce upregulation of UPS, atrogin-1, and MuRF1, which may lead to muscle protein degradation [24].

### 2.5. Metabolic Acidosis

Metabolic acidosis is a universal feature in the majority of patients diagnosed with CKD who have a glomerular filtration rate (GFR) that has decreased to less than 20% to 25% of the normal rate. Metabolic acidosis has been shown to cause negative nitrogen balance and decrease albumin synthesis, leading to muscle wasting. In addition, metabolic acidosis causes muscle protein degradation by activation of the UPS and caspase-3, and reduced intracellular pH in muscle cells impairs PI3K and p-Akt signaling [14, 25–27]. Moreover, decreased growth hormone concentration, low IGF-1 level, and increased glucocorticoids production have been reported in individuals with metabolic acidosis, which may partly explain the high prevalence of muscle wasting in CKD patients [28, 29].

### 2.6. Sex Hormones

It is well known that both estrogen and testosterone affect protein synthesis and degradation and that testosterone produces a more prominent effect on muscle protein turnover than does estrogen. A low testosterone level can induce muscle protein degradation by impaired IGF-1 signaling and promote muscle catabolism by upregulation of myostatin expression [30]. Evidence shows that low-testosterone concentrations are highly prevalent in elderly people and CKD patients and that low-testosterone levels closely correlate with muscle wasting and mortality in HD patients [31, 32]. Thus, androgen deficiency may be involved in the complex mechanisms that underlie muscle wasting in CKD patients.

## 3. Therapeutic Frontiers of Muscle Wasting in Hemodialysis Patients

To improve the quality of life and the long-term prognosis of patients, development of effective therapeutic strategies for muscle wasting is necessary in HD patients. Recent advances in understanding of the molecular mechanisms involved in CKD-related muscle wasting provide new hope for the development of a set of new therapies. The following therapeutic interventions have been reported to be effective in the improvement of muscle strength in CKD patients (Table 1).

### 3.1. Endurance and Resistance Exercise

Endurance (aerobic) and resistance (anaerobic or strength training) exercise have been reported to reduce muscle wasting in HD patients. Resistance exercise induces muscular contraction, which may increase the strength, anaerobic endurance, and the size of skeletal muscles. Resistance exercise may be divided into traditional power lifting and Olympic lifting. Both endurance exercise and resistance exercise can lead to increased skeletal muscle strength and power [33].

In a typical endurance exercise program, at least 30 minutes per day of moderate intensity exercise must be performed 5 days per week. In patients who perform poorly, such as HD patients, it is necessary to initiate exercise training with a lower intensity, shorter duration, and fewer days per week [34, 35]. In patients on maintenance HD, a considerable improvement of aerobic exercise capacity and muscle strength has been demonstrated after endurance training [36]. It has been shown that after 6 months of supervised endurance exercise, an increase in ejection fraction, cardiac output index, and systolic volume index occurs [37]. An intradialytic aerobic exercise program with exercise bicycles in HD patients correlated with a considerable reduction in intra- and interdialytic systolic and diastolic blood pressure. In addition, 4 months of aerobic training in predialysis patients also leads to a major reduction of systolic and diastolic blood pressure, accompanied by a considerable reduction in the number of prescribed antihypertensive drugs [38, 39]. Moreover, improvement of insulin resistance and anorexia have been reported in HD patients who have undergone endurance exercise training. It is noteworthy that the positive effects yielded from these exercise programs were completely reversed 2 months after training ceased [40, 41].

A resistance exercise program should be performed gradually and at least twice a week and should include training for all the major muscle groups. In HD patients with impaired exercise capacity and marked muscular atrophy, resistance exercise reduces muscle wasting and increases muscle fibers, based on histological examinations [42]. The serum levels of inflammatory factors, such as C-reactive protein and interleukin-6, are reported to decrease after 12 weeks of resistance exercise [43].

### 3.2. Treatment of Insulin Resistance

Insulin resistance induces muscle wasting through complex mechanisms, including insulin/IGF-1 and PI3K/Akt signaling pathways. Improving insulin resistance is crucial to prevent muscle wasting in patients diagnosed with HD. Aerobic exercise in HD patients is effective in improving insulin resistance [40]. Thiazolidinediones, which are insulin sensitizers, are widely used in the treatment of type 2 diabetes and have been shown to improve insulin resistance through activation of the PI3K/Akt pathway by initiating IRS signaling. Because the catabolism of thiazolidinediones mainly occurs in the liver, it is a potential drug for improving insulin resistance in HD patients [44–46]. Thiazolidinedione should be administered with caution because of increased risks of cardiovascular events and bladder cancer [47]. However, limited human data supports the major role of insulin sensitizers on muscle wasting in HD patients.

### 3.3. Correction of Metabolic Acidosis

Metabolic acidosis is an inevitable condition in patients with CKD, particularly those with HD [48–50]. A sodium bicarbonate (NaHCO_3_) supplement has been demonstrated to improve growth in infants and children with acidosis [51]. In addition, protein loss in the muscle is approximately 2-fold higher in patients with serum NaHCO_3_ levels <16 mM as compared to those with levels >22.6 mM [52]. These data suggest that maintenance of the serum NaHCO_3_ level >22.6 mM may be a therapeutic goal for reducing muscle wasting in HD patients with metabolic acidosis.

### 3.4. Hormone Therapy

A decreased serum testosterone level has been frequently encountered in patients diagnosed with advanced CKD and on maintenance HD, and impaired IGF-1 signaling may participate in the mechanism of androgen-deficiency-mediated muscle wasting. In men with hypogonadism, testosterone supplements for 12 weeks improve muscle mass and strength [53, 54]. In HD patients, body composition and physical function improve considerably after treatment with an anabolic steroid, 19-nortestosterone (nandrolone decanoate) [55]. Administration of 100 mg nandrolone per week for 24 weeks increases lean body mass approximately 2-fold. Although nandrolone decanoate is effective in improving muscle wasting, its side effects, including gynecomastia, erectile dysfunction, and increased cardiovascular risks, should be cause for caution [56].

### 3.5. Nutrition

In HD patients, malnutrition is a major problem caused by anorexia and hypercatabolism through complex mechanisms including inflammation, metabolic acidosis, insulin resistance, and uremic toxins [57]. Anorexia is a common manifestation of uremic syndrome and is associated with increased risks of mortality and hospitalization in HD patients [58]. Appetite stimulants, such as megestrol acetate, melatonin, thalidomide, ghrelin, and cyproheptadine, are potential therapeutic agents for resolving anorexia in HD patients. Cyproheptadine is a first-generation antihistamine that generates additional anticholinergic and antiserotonergic effects. Although cyprohetadine is used as an appetite stimulant in children diagnosed with HD [59], its role in adult HD patients is not well established. Megestrol acetate is a steroidal progestin and progesterone derivative exhibiting antiandrogenic and antiestrogenic effects and has been proven to be an effective appetite stimulant in patients with advanced cancers. In patients on maintenance HD, megestrol acetate has also been reported to be useful to improve anorexia and muscle wasting. Oral administration of 160 mg megestrol acetate daily for 2 months considerably increases body mass index and serum albumin levels [60, 61]. The side effects of megestrol acetate, including impotence, hypogonadism, and increased risk of thromboembolism, should be monitored closely. No large scale clinical trials have been conducted to define the therapeutic effects of these agents in HD patients.

In addition to appetite stimulants, direct nutritional supplements are essential to reduce muscle wasting. Oral, enteral, or parenteral nutritional supplements should be considered if unresolved anorexia occurs. A systematic review and meta-analysis of 18 studies indicated that enteral nutritional supplements in HD patients resulted in increased total energy and protein intake and elevated serum albumin levels by 0.23 g/dL [62]. Oral nutrition alone and combined with intradialytic parenteral nutrition in patients diagnosed with HD revealed similar results, including improvement in body mass index, elevated serum albumin and prealbumin levels, decreased 2-year mortality, and reduced hospitalizations [63–65].

## 4. Conclusion

Muscle wasting in HD patients is caused by complex mechanisms and agents, including UPS, caspase-3, insulin/IGF-1, glucocorticoid, metabolic acidosis, and sex hormone-related signaling pathways. Development of new drugs targeting UPS, caspase-3, and insulin/IGF-1 offers new hope for the treatment of muscle wasting. Correction of metabolic acidosis with sodium bicarbonate reduces muscle protein degradation. Megestrol acetate and nandrolone decanoate are clinically available and could be applied to reduce muscle wasting. Adequate nutritional supplements are vital because they may improve muscle mass and reduce mortality. Endurance exercise not only reduces muscle wasting, but also improves cardiac ejection fraction, blood pressure, and insulin resistance. Recent advances in understanding the molecular mechanisms of muscle wasting provide opportunities to resolve this clinical problem.

## Figures and Tables

**Table 1:** Therapeutic interventions for muscle wasting in hemodialysis patients.

Therapeutic targets	Therapeutic interventions
Muscle strength	Endurance exercise and resistance exercise
Insulin resistance	Endurance exercise, insulin sensitizers (e.g., Thiazolidinediones)
Metabolic acidosis	NaHCO_3_ supplement
Hypogonadism	Testosterone supplement (e.g., Nandrolone decanoate)
Malnutrition	Endurance exercise, nutritional supplements, megestrol acetate (for adult) cyprohetadine (for children)
